# Effect of the Interrelation between CYP3A5 Genotype, Concentration/Dose Ratio and Intrapatient Variability of Tacrolimus on Kidney Graft Function: Monte Carlo Simulation Approach

**DOI:** 10.3390/pharmaceutics13111970

**Published:** 2021-11-20

**Authors:** Nikola Stefanović, Radmila Veličković-Radovanović, Katarina Danković, Ivan Pavlović, Aleksandra Catić-Đorđević, Jelena Bašić, Milena Despotović, Tatjana Jevtović-Stoimenov, Branka Mitić, Tatjana Cvetković

**Affiliations:** 1 Department of Pharmacy, Faculty of Medicine, University of Nis, 18000 Nis, Serbia; aleksandra.catic@medfak.ni.ac.rs; 2 Department of Pharmacology with Toxicology, Faculty of Medicine, University of Nis, 18000 Nis, Serbia; radavel@medfak.ni.ac.rs; 3 Clinic of Nephrology, University Clinical Center Nis, 18000 Nis, Serbia; branka.mitic@medfak.ni.ac.rs; 4 Faculty of Medicine, University of Nis, 18000 Nis, Serbia; ketrin92pk@gmail.com; 5 Faculty of Mechanical Engineering, University of Nis, 18000 Nis, Serbia; ivanp79@gmail.com; 6 Department of Biochemistry, Faculty of Medicine, University of Nis, 18000 Nis, Serbia; jelena.basic@medfak.ni.ac.rs (J.B.); m-despotovic@outlook.com (M.D.); tatjana.jevtovic.stoimenov@medfak.ni.ac.rs (T.J.-S.); tatjana.cvetkovic@medfak.ni.ac.rs (T.C.); 7 Department of Internal Medicine, Faculty of Medicine, University of Nis, 18000 Nis, Serbia; 8 Center for Clinical and Medical Biochemistry, University Clinical Center Nis, 18000 Nis, Serbia

**Keywords:** intrapatient variability, kidney transplantation, CYP3A5 genotype, tacrolimus, therapeutic drug monitoring

## Abstract

**Background**: Tacrolimus (Tac) is characterized by large between- and within-patient (IPV) variability in pharmacokinetics and exposure. **Aim**: This study aimed to assess and validate the effect of Tac IPV and trough concentration-to-dose ratio (C_0_/D) over 6–12 months on reduced estimated glomerular filtration rate (eGFR) values in the late period after kidney transplantation (Tx), applying Monte Carlo (MC) simulation. **Methods**: The previously published linear regression was the basis for MC simulation, performed to determine how variations in significant predictors affect the distribution of eGFR from 13 to 36 months post-transplantation. The input C_0_/D values were derived from CYP3A5 genotype subgroups. **Results**: Patients characterized by high Tac IPV and low mean C_0_/D over 6–12 months could have been at greater risk of lower eGFR values in a three-year period following Tx compared to the other patient groups. This effect was more pronounced in patients with a lower eGFR at the 6th month and a history of acute rejection. The proven contribution of CYP3A5 expresser genotype to low C_0_/D values may suggest its indirect effect on long-term graft function. **Conclusion**: The findings indicate that simultaneous assessment of Tac IPV, C_0_/D, and CYP3A5 genotype may identify patients at risk of deterioration of graft function in the long-term post-transplantation period.

## 1. Introduction

Tacrolimus (Tac) is the backbone of the most immunosuppressive protocols after kidney transplantation (Tx) nowadays, and it will probably continue to be within the next decade. Still, Tac is characterized by large between- and within-patient (intra-patient, IPV) variability in pharmacokinetics and exposure [1,2]. Tacrolimus trough concentration (C_0_)-to-dose ratio (C_0_/D) is assumed to be an adequate parameter for estimating the between-patient pharmacokinetic variability, whereby Tac IPV is defined as a fluctuation of C_0_ over a certain period of time, during which Tac daily dose remains constant [2]. Recent studies have indicated that Tac C_0_/D, mostly determined at the 3rd month post-transplantation, could be a surrogate marker for Tac metabolism rate and exposure [2,3]. In addition, C_0_/D is strongly affected by the cytochrome P450 (CYP) 3A5 (6986A>G) genotype, whereas the carriers of CYP3A5*1 allele require a higher Tac daily dose (TDD) to achieve the target C_0_ range [4,5]. It has been assumed that low Tac C_0_/D, which corresponds with a faster metabolism and lower bioavailability, and high Tac IPV within the first post-transplantation year may be associated with kidney graft impairment or loss in the late period after Tx [6,7].

The present study is the continuation of the research published by Stefanović et al. [8], whereby the proposed linear regression model revealed the significant and independent predictors of estimated glomerular filtration rate (eGFR) values up to three years after Tx: established eGFR at 6th month (eGFR6), occurrence of acute rejection episodes within the first-year post-transplantation (AR), Tac IPV, and mean C_0_/D during 6–12 months after Tx.

The evaluation of Tac IPV or C_0_/D only may not be sufficient in clinical practice, which is why other clinical parameters that were identified to correlate with long-term graft function should be also observed. In order to overcome a small study population, lack of power, and risk of causing statistical errors, we performed Monte Carlo (MC) simulation using a large hypothetical cohort of patients to demonstrate which combination of model parameters would exhibit the greatest detrimental effect on the graft function. Given that linear regression analysis is a baseline probability analysis, which can give insight into the relative contribution of individual predictors to the overall outcome and direction of their effects, this analysis serves as a basis for applying a more sophisticated decision-making MC method [9]. Therefore, the aim of this study was to assess and validate the effect of Tac IPV and C_0_/D over 6–12 months on reduced eGFR values in the late period after Tx, applying the MC simulation method. In addition, the assessment of Tac IPV and C_0_/D impact on eGFR was performed on four different occasions with respect to AR and eGFR6. Additionally, the study examined the effect of the CYP3A5 genotype on Tac C_0_/D within the first post-transplantation year.

## 2. Materials and Methods

### 2.1. Study Population

The pharmacokinetic/pharmacogenetic study was conducted at the Research Centre for Biomedicine, Faculty of Medicine, University of Nis and the Clinic of Nephrology, University Clinical Center Nis, Serbia. The study protocols were approved by the Ethics Committee of Faculty of Medicine, University of Nis (No 12-2307-2/5 from 3 October 2016. and No 12-6972-2/5 from 7 February 2018). The study included 103 Caucasian patients who underwent the first transplantation surgery, and data obtained from 2530 patients’ examinations in the period between 2008 and 2016. In most cases, the primary kidney disease was glomerulonephritis, followed by hypertensive nephropathy, diabetic nephropathy, or vesicoureteral reflux, or the cause was unknown. In addition, a few patients had pyelonephritis, IgA nephropathy, Alport syndrome or polycystic kidney disease. The kidney transplant recipients met some exclusion criteria before they were enrolled in this study: patients who underwent Tx less than 3 years ago, with any sign of graft failure demanding regular dialysis within the first post-transplantation year and with uncontrolled hypertension. In addition, only patients with low immunological risk were eligible for enrolment.

### 2.2. Study Design

**Figure 1:**
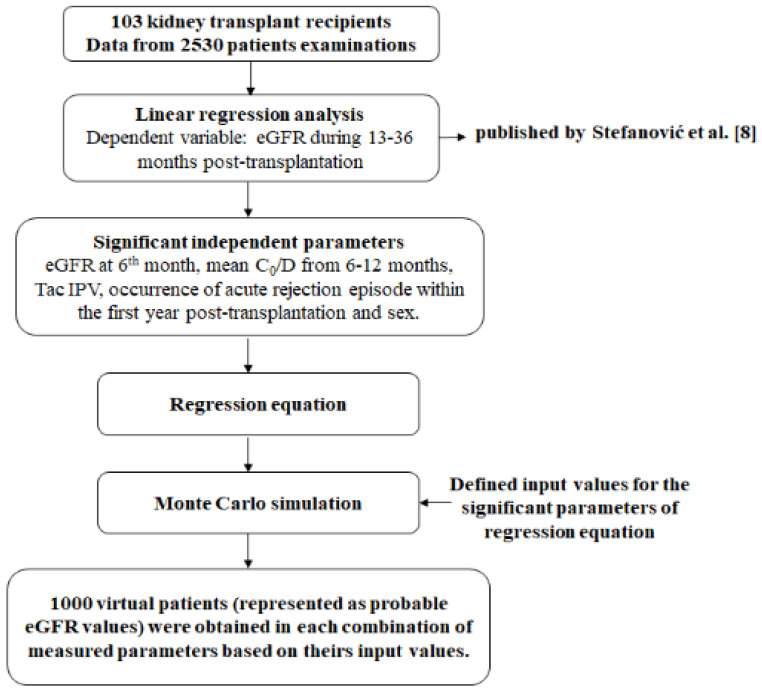
Study design.

Multivariable linear regression analysis was conducted in order to estimate factors influencing eGFR values from 13 to 36 months after Tx. With reference to the performed experimental study [8], where 103 patients were included, a regression equation was created, which served as a basis for MC simulation. All of the significant parameters from the regression analysis were assigned input values, defined by upper and lower limits for each of the measured parameters. The limits were determined by calculating mean values and standard deviation of significant parameters or by clinical relevance. Calculated limits gave a very high probability that the measured values of any other patient would be in that range. Further, according to upper and lower values for each measured parameter, 1000 new values were generated from these ranges. Therefore, numerical values obtained in the previous step (1000 numerical values of significant parameters were obtained) were then randomly combined and used as input parameters in the simulation, which generated 1000 numerical patients’ parameters and outcomes’ values determination (Figure 1). Therefore, the MC method includes mathematical models and numeric simulations that can ultimately provide the prediction of potential long-term outcomes after Tx, based on the derived regression equation. Monte Carlo simulation repeatedly simulates the model, each time drawing a different random set of values (inputs) from the sampling distribution of the model parameters in order to produce distributions of possible outcome values (outputs) [10,11].

### 2.3. Immunosuppressive Protocol

Patients were prescribed triple immunosuppressive protocol, which besides Tac included mycophenolate mofetil (MMF) or mycophenolic acid (MPA) and corticosteroid, mostly prednisone. All patients started with intravenous methylprednisolone with an initial dose of 0.5 g/day, which was later switched to prednisone (initial dose of 1 mg/kg/day), MMF, 1.5–2 g/day or MPA, 1080–1440 mg/day orally, and 20 mg of monoclonal antibody basiliximab, which was administered at the first and the fourth day after Tx. The first oral Tac dose was administered on day 5 post-transplantation at 8:00 a.m. before breakfast (0.1 mg/kg or 0.2 mg/kg). Considering Tac pharmaceutical formulation, 78 patients were prescribed conventional preparation, immediate-release hard capsules (Prograf^®^, Astellas Ireland Co. Ltd., Astellas, Ireland), twice-a-day formulation (Tac-TD), whereas 25 patients were prescribed prolonged-release hard capsules (Advagraf^®^, Astellas Ireland Co. Ltd.), once-a-day formulation (Tac-OD). Furthermore, Tac was adjusted according to the therapeutic drug monitoring (TDM) in order to achieve a target therapeutic range of 8–12 ng/mL for the first 90 days and 6–10 ng/mL afterward. Patients also received antihypertensive drugs: beta blockers (bisoprolol, carvedilol, or metoprolol) and/or calcium channel blocker (amlodipine or felodipine) in monotherapy as well as in the combination. In addition, some patients received angiotensin-converting enzyme (ACE) inhibitors (fosinopril and zofenopril), methyldopa, or furosemide. All patients had proton pump inhibitor (PPI) in their treatment protocol. Detailed information regarding biochemical monitoring can be found in Stefanović et al. [8]. The Modification of Diet in Renal Disease (MDRD) equation was used for the calculation of eGFR [12]. Data regarding AR and delayed graft function (DGF) were obtained from medical records. Biopsies were performed at cause only.

### 2.4. Pharmacokinetic Data

Tacrolimus daily dose and C_0_ were recorded during the entire observation period of three years post-transplantation. Dosage regimen was obtained from the medical records, while C_0_ was measured by chemiluminescent microparticle immunoassay (CMIA) method according to the manufacturer’s instructions (Architect, Abbott, Abbott Park, IL, USA). Tacrolimus C_0_/D was calculated as C_0_ divided by the corresponding TDD. Tacrolimus IPV was calculated as the coefficient of variation (CV%) of the C_0_/D between 6 and 12 months after Tx (formula):

(1) $$\mathrm{CV}\%=\frac{\bar{x}}{\mathrm{SD}}\times 100\%$$

where $\bar{x}$ is the mean C_0_/D of available samples during 6–12 months after Tx, calculated for each patient; SD is the standard deviation of available samples over 6–12 months after Tx, calculated for each patient; average number of C_0_ used for IPV calculation was 7 per patient, range 4–11.

### 2.5. Genotyping CYP3A5

CYP3A5 6986A>G (rs776746) genotyping was performed using TaqMan^®^ Drug Metabolism Genotyping Assays for CYP3A5*3 (C__26201809_30) (Applied Biosystems, Carlsbad, CA, USA) on the Mx3005P Real-Time PCR System (Agilent Technologies, Santa Clara, CA, USA), according to the manufacturer’s instructions. Previously, DNA was extracted from the whole blood (200 µL) with EDTA as an anticoagulant using Genomic DNA Purification Kit (Fermentas, Thermo Scientific, Vilnius, Lithuania) according to the manufacturer’s instructions.

### 2.6. Statistical Analysis

The characteristics of the study group were expressed as median and interquartile range or number and frequency (%). Mann–Whitney U test (not normally distributed data) was employed for the comparison of TDD, C_0_, and C_0_/D with respect to the CYP3A5 genotype. In addition, Mann–Whitney U test was used to compare eGFR, Tac IPV, and C0/D between Tac formulation, whereas Chi square test was performed to compare the frequency of AR and presence of CYP3A5*1/*3 genotype. Linear regression analysis was performed to evaluate the potential influence of independent predictors on eGFR. All analyses were performed with SPSS statistical analysis software, version 20.0 (SPSS, Chicago, IL, United States) at the significance level set at *p* < 0.05. MATLAB R2017b (MathWorks) software was used to perform the MC simulation.

## 3. Results

The characteristics of the patient population are given in Table 1.

Furthermore, due to study heterogeneity considering the Tac formulation, Table 2 shows relevant pharmacokinetic and clinical parameters in relation to drug formulation.

**Figure 2:**
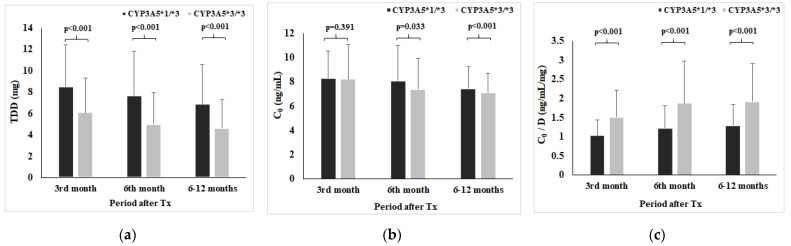
Tacrolimus daily dose (**a**), C_0_ (**b**), and C_0_/D (**c**) with respect to CYP3A5 genotype.

Figure 2 shows TDD (a), C_0_ (b), and C_0_/D (c) with respect to CYP3A5 genotype at 3rd, 6th, and 6–12 months post-transplantation. Therefore, CYP3A5*1/*3 genotype carriers had lower Tac C_0_/D than CYP3A5*3/*3 carriers in all observed time points (at 3rd month: 1.04 ± 0.39 vs. 1.52 ± 0.70, Z = −11.729, *p* < 0.001; at 6th month: 1.23 ± 0.58 vs. 1.88 ± 1.10, Z = −5.723, *p* < 0.001; period 6–12 months: 1.30 ± 0.54 vs. 1.92 ± 0.98, Z = −10.738, *p* < 0.001).

**Figure 3:**
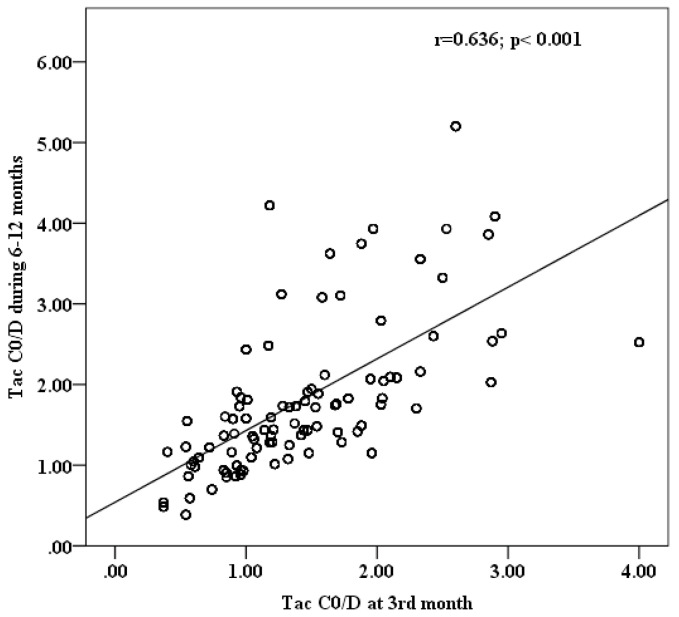
Correlation analysis between Tac C_0_/D at 3rd and mean C_0_/D at 6–12 months post-transplantation.

Since the performed regression analysis and MC stimulation included mean C_0_/D values at 6–12 months as predictor and input variable, respectively, an accompanying analysis demonstrates a high positive correlation between Tac C_0_/D at the 3rd month (indicative of poor post-transplantation outcomes) and mean C_0_/D values at 6–12 months after Tx (Figure 3).

After obtaining statistically significant predictors in multivariate regression analysis (Table 3), i.e., eGFR6, Tac IPV, Tac C_0_/D, sex, and AR, a regression equation was constructed based on the coefficients and causal relationship between the dependent variable and independent predictors, which represents a basis for the MC simulation (Model*). The covariate coefficients in Model* slightly differ from the published model [8] due to different presentations of Tac IPV covariate, but this change was not significant for further analysis.

IPV—intrapatient variability; C_0_/D—dose-adjusted trough concentration

(2) $$y=11.256+0.764\left(\mathrm{eGFR}6\right)–0.103\left(\mathrm{TacIPV}\right)+1.439\left(\mathrm{Sex}\right)\;\;\;\;\;\;\;\;\;\;\;\;\;\;\;\;+1.676\left(C_{0}/\mathrm{Dduring}\;6-12\;\mathrm{months}\right)–10.112\left(\mathrm{AR}\right)\left[\mathrm{Model}*\right]$$

where y stands for eGFR between 13 and 36 months post-transplantation.

For the purpose of the present research, an MC simulation was performed based on the Model*. The MC simulation was carried out with the goal to determine how variations in predictors affect the distribution of eGFR values from 13 to 36 months after Tx. In addition, this study aimed to estimate a contribution of Tac IPV, C_0_/D, and CYP3A5 genotype, defined by C_0_/D values in CYP3A5 genotype subgroups, impact on eGFR in different clinical situations. In this way, by using the MC method, we have obtained 1000 values of eGFR between 13 and 36 months, which represented the most expected values of eGFR in this study. The input values of independent variables used for MC simulation are present in Table 4.

The continuous variables, eGFR6 (I: 30–44 mL/min/1.73 m^2^, II: 45–59 mL/min/1.73 m^2^) and Tac IPV (I:15–29.99%, II:30–59.99%), were categorized in two subsets regarding their probability of distribution. As for Tac C_0_/D, randomly selected sets of values within one standard deviation of the mean value were used for the simulation. Since the CYP3A5 genotype was excluded from the multivariable analysis due to the correlation with C_0_/D, it was implemented in the simulation through different calculations for C_0_/D in CYP3A5*1/*3 and CYP3A5*3/*3 genotype carriers.

**Figure 4:**
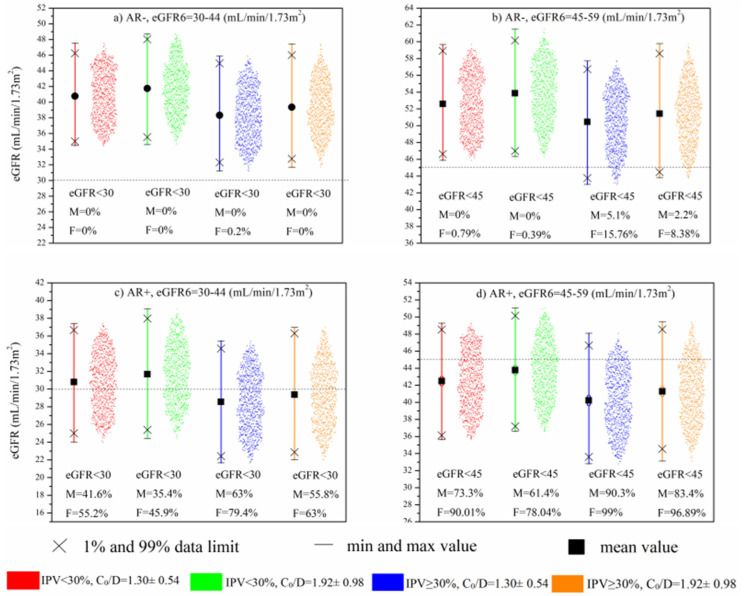
MC simulation: Impact of eGFR6, Tac IPV, and mean C_0_/D during 6–12 months after Tx on long-term kidney function in patients without AR (**a**,**b**) and with AR (**c**,**d**).

Figure 4a,b demonstrates the contribution of eGFR6, Tac IPV, and Tac C_0_/D to the eGFR values between 13 and 36 months in cases without AR. It can be seen that the kidney transplant recipients with a combination of higher Tac IPV values (>30%) and lower Tac C_0_/D were more likely to have lower eGFR values than the other combinations. Although none of the patients shown in Figure 4a reached eGFR below < 30 mL/min/1.73 m^2^, high IPV/low C_0_/D patients tended to show group eGFR values at the lower data limit.

When the simulation model included AR as a predictor (Figure 4c,d), the negative impact of high Tac IPV and low Tac C_0_/D was amplified. Specifically, in cases with worse baseline kidney function (eGFR6: 30–44 mL/min/1.73 m^2^), it can be seen that the aforementioned combination generated 79.4% and 63% eGFR simulations below 30 mL/min/1.73 m^2^ in females and males, respectively (Figure 4c). The significance of this result is reflected in the fact that kidney transplant recipients with the opposite combination of lower Tac IPV (15–29.99%) and higher Tac bioavailability tended to have simulated eGFR values below 30 mL/min/1.73 m^2^, almost twice as low as the high-Tac IPV and low-Tac bioavailability group. Figure 4d demonstrates that in patients with better baseline kidney function (eGFR6: 45–59 mL/min/1.73 m^2^), there was still a tendency toward lower eGFR values, resulting in a large number of simulations below 45 mL/min/1.73 m^2^, with particular emphasis again on high Tac IPV and low Tac C_0_/D group (99% and 90.3% of simulations in females and males, respectively).

## 4. Discussion

In recent years, research and development in transplantation medicine has been focused on the improvement of long-term kidney transplant outcomes. Since there have been limited novel immunosuppressive drugs, optimization of current immunosuppressive protocols based on Tac is of the utmost importance [13]. Therefore, this study tried to enlighten the potential effect of Tac IPV and between-patient pharmacokinetic variability (defined as C_0_/D) within the first post-transplantation year on the graft function in the long-term period after Tx.

It was previously demonstrated that high Tac IPV [7,14,15,16] or low C_0_/D [3,6,17] may cause eGFR decline, graft rejection, or loss in the late post-transplant period. Still, it has not been entirely elucidated whether or not these parameters should be considered simultaneously. Kuypers DR suggested that the effect of Tac IPV may differ remarkably with regard to Tac exposure [18]. In addition, patients with constantly low or high Tac exposure (Tac C_0_) may be more prone to unwanted transplantation outcomes. A significant between-patient (20–60%) and within-patient (10–40%) variability in Tac C_0_ [19] requires regularly applied TDM and dose adjustments to maintain its value within the target therapeutic range. Therefore, C_0_/D could be a better parameter for proper comparison of Tac exposure between patients. In addition, lower exposure leads to higher dose requirements (low C_0_/D), which may be associated with Tac nephrotoxic effects due to increased exposure early after drug administration [20]. As was said in the introduction, the present study is the continuation of previous research [8], which showed that Tac IPV, mean Tac C_0_/D over 6–12 months, AR, and eGFR6 significantly and independently affected eGFR between the 1st and the 3rd post-transplantation year.

Given that the use of standard statistical methods bears a certain degree of bias and uncertainty, simulation methods enable the prediction of a set of outcomes based on an estimated range of values as opposed to the set of fixed input values. In this way, the MC simulation enables the confirmation of linear regression analysis results and provides more reliable evidence of the objective contribution of the parameters of interest [21].

Hence, based on the input parameters that were chosen, MC simulation indicated that patients with high IPV and low C_0_/D of Tac in the first post-transplantation year had the most deleterious effect on the late eGFR values. Still, this effect was additionally amplified in patients with a history of AR. Although most of the patients with eGFR6 within 45–59 mL/min/1.73 m^2^, history of AR, and combination of high IPV/low C_0_/D would have reached eGFR < 45 mL/min/1.73 m^2^ between the 1st and the 3rd post-transplantation year, none of them would have reached eGFR < 30 mL/-min/1.73 m^2^ (Figure 4d). Conversely, the group of patients with the worse eGFR6, 30–44 mL/min/1.73 m^2^, present with significantly more cases with eGFR < 30 mL/min/1.73 m^2^, indicating that the functional status of the graft early after transplantation is a strong contributing factor to the effect of Tac inter- and intrapatient pharmacokinetic variability. In addition, almost twice as many patients with the combination of high IPV/low C_0_/D achieved eGFR < 30 mL/min/1.73 m^2^ compared to the low IPV/high C_0_/D patients’ group. This result is in the accordance with Sablik et al.’s findings, which showed a comparable Tac IPV between chronic active antibody-mediated rejection (c-ABMR) patients and controls. Although high Tac IPV per se does not predispose patients to the development of c-aABMR, it was associated with inferior graft survival once c-ABMR was diagnosed [22]. Kim et al. suggested that high-Tac IPV significantly increases the risk of graft loss and antibody-mediated rejection in the late period after Tx, especially in patients with high immunological risk [23].

Tacrolimus C_0_/D within the first year post-transplantation is assumed to be a surrogate marker for drug metabolism rate [17]. The majority of published studies indicated an association between C_0_/D level at the 3rd month post-transplantation with late eGFR reduction or graft and patient survival [7,17,20]. Considering C_0_/D at the 3rd month, patients were categorized into three groups: <1.05 ng/mL/mg were fast metabolizers, 1.05–1.54 ng/mL/mg were intermediate metabolizers, and ≥1.55 ng/mL/mg were slow metabolizers [20,24]. Hence, a correlation analysis was performed and results indicated a strong positive association between C_0_/D over 6–12 months and C_0_/D at the 3rd month post-transplantation. Furthermore, Nowicka et al. demonstrated that C_0_/D at the 6th month post-transplantation may be useful to categorize patients with respect to the risk of developing deteriorated graft function in a 2-year follow-up. As a matter of fact, fast metabolizers (C_0_/D ratio <1.47 ng/mL/mg) had significantly worse graft function throughout the whole study period (*p* < 0.05 at each time point) and were significantly less likely to develop or maintain a good graft function (≥45 mL/min/1.73 m^2^) compared to slow metabolizers [25]. Since there was a strong correlation between the CYP3A5*1/*3 genotype and lower average C_0_/D, the obtained results indicated an indirect association between CYP3A5 genotype and eGFR decline. It has been well documented that CYP3A5 genotype is the strong predictor of C_0_/D values [5,26,27]. Still, there have been no conclusive data considering a negative influence of CYP3A5*1 allele on kidney allograft, despite it being shown for C_0_/D [20]. A potential explanation for such a phenomenon can be found in the lower prevalence of CYP3A5*1 allele in European populations compared to others [28]. The proven contribution of CYP3A5*1 genotype to the low C_0_/D values requires the close monitoring of C_0_/D levels in patients carrying the CYP3A5 expresser genotype. Although the presence of CYP3A5*1 allele and low C_0_/D values seem to be overlapping factors, considering lower bioavailability and higher Tac dose requirements, the impact of C_0_/D on long-term post-transplantation outcomes is stronger [20]. It is most likely that other factors (inhibitor and inducers of CYP3A, hematocrit, etc.) may contribute to inter-individual variability of Tac. Therefore, patients with CYP3A5*3/*3 genotype had C_0_/D values assigned to the CYP3A5*1/*3 subgroup, but most of the patients with CYP3A5*1/*3 genotype will have lower C_0_/D values. Monte Carlo simulation was performed to assess the influence of CYP3A5 genotype on the late kidney graft function, through expressers’ and non-expressers’ C_0_/D values at 6–12 months post-transplantation.

It was proposed that high-Tac IPV might have been related to the development of donor-specific antibodies and consequently graft loss [29,30]. Nevertheless, high Tac IPV may also be indicative of the chronic histologic lesions before any evidence of graft dysfunction. Specifically, Vanhove et al. found that patients with high-Tac IPV had a significantly increased risk of occurrence of moderate to severe fibrosis and tubular atrophy compared to patients with low-Tac IPV, which was confirmed by paired protocol biopsies at the 3rd month and 2nd year post-transplantation [31].

Accordingly, Tac bioavailability (C_0_/D) should be considered alongside Tac IPV. Lower Tac C_0_/D is associated with lower drug exposure and consequently higher dose requirements to achieve or maintain the target therapeutic level. Furthermore, Thölking et al. showed that higher doses led to higher peak levels within the first hours (C_2_) upon oral administration [32]. Accordingly, lower bioavailability is instead associated with acute calcineurin inhibitor nephrotoxicity, and development of BK virus infection and BK-virus is associated nephropathy than the development of donor-specific antibodies [24]. Therefore, determination of low Tac C_0_/D was not enough to elucidate whether underexposure or higher C_2_ levels are indicative of kidney graft impairment. However, determination of Tac C_2_ is not standard clinical practice, and we did not perform that analysis. Nevertheless, acute conditions (rejection) per se is an important predictor of adverse Tx outcomes, whereby management of these episodes can also provoke high IPV [33]. Still, it may be of great importance whether higher peak levels occur within the high-Tac IPV patient group or not. Furthermore, CYP3A5 genotyping in clinical practice could be advisable due to the identification of individuals who potentially strive for low C_0_/D values, as was shown. However, CYP3A5 genotyping is not standard practice in transplantation medicine.

Previously, it was shown that the combined effect of Tac high IPV/low C_0_/D over 6–12 months led to significantly more composite endpoints within three years post-transplantation compared to other combined groups [34]. Besides the present paper confirming those findings, it additionally suggests that the combined effect was more pronounced in patients who already had diminished kidney function. Our results rely on the finding of Hariharan et al. in light of recognition of the notable impact of graft function within the first year post-transplantation on long-term graft function [35]. Of note is also the obtained difference between females and males; i.e., the simulation model in females produced notably lower eGFR values, which can partly be explained by baseline gender-based difference in eGFR.

Some limitations of the study need to be mentioned. The first drawback of the present research is its retrospective study design. Hence, it was not possible to examine the factors that affect Tac IPV, such as lack of adherence or the use of concomitant medications. In addition, the present study is heterogeneous regarding Tac formulations, which can be assumed as one of its limitations. Although the lack of adherence is largely recognized as a significant contributor to the high IPV, there is an insufficient number of studies that examined patients’ adherence in relation to IPV (even with conflicting results) [36].

Giza et al. showed that Tac IPV could be influenced by the number of concomitant medications. The authors demonstrated an increasing trend for median IPV proportional to the increasing number of medications. Still, the study did not show a significant difference in Tac IPV in patients treated with Tac-TD or Tac-OD [37]. Although we did not compare Tac IPV in relation to the number of concomitant medications, the results of our study were in accordance with Giza et al. considering Tac formulations and IPV. Conversely, some previous studies showed differences in Tac IPV after conversion from Tac-TD to Tac-OD [38,39]. Stifft et al. concluded that intrapatient CV of the Tac area under the curve from 0 to 24 h (AUC0–24) was improved after Tac-TD conversion to Tac-OD in stable kidney transplant recipients. Still, patient groups did not differ in intrapatient CVs of Tac C_0_, which is in accordance with our study. Stifft et al. suggested that the concrete difference was most likely due to the different intrinsic pharmacokinetic properties of Tac formulations. Furthermore, the authors showed that the intrapatient CV of Tac AUC0–24 was especially noticed in CYP3A5 expressers. The same finding was not shown for the intrapatient CV of Tac C_0_ in relation to the CYP3A5 genotype. Most of the previous studies did not find the association between Tac IPV and CYP3A5 genotype [1]. Wu et al. demonstrated a significant reduction in Tac IPV after conversion from Tac-TD to Tac-OD. The authors believe the Hawthorne effect may be a potential explanation for such a finding, whereby subjects improve or modify an aspect of their behavior that is being experimentally measured (e.g., compliance) due to the fact they are being studied. Still, both studies (Stifft et al. and Wu et al.) investigate Tac IPV after Tac-TD vs. Tac-OD conversion, while our study and the study of Giza et al. included de novo kidney transplant recipients on Tac-TD or Tac-OD formulation [37,38,39].

Furthermore, other limitations of the study need to be pointed out, such as the small number of patients included in regression analysis and the single-center setting. The limitations that would potentially arise from a small number of patients were generally overcome by a large number of data per patient and the use of MC simulation. Specifically, one of the main advantages of the MC method in this work is that the initial tested sample was numerically significantly extended in its goal to calculate the most of possible simulation output values (i.e., outcomes). On the other hand, the single-center design enables the close monitoring of patients and the application of timely patient education.

## 5. Conclusions

In conclusion, the MC simulation study showed that patients who were characterized by high Tac IPV and lower mean C_0_/D over 6–12 months post-transplantation may have been at greater risk of lower eGFR values in the three-year period following Tx compared to the other groups (i.e., high IPV/high C_0_/D, low IPV/low C_0_/D, low IPV/high C_0_/D). This effect was more pronounced in patients with a worsening graft function in the first year after Tx, including a history of AR. In addition, the carriers of CYP3A5*1/*3 genotype tended to have lower C_0_/D values compared to the CYP3A5*3/*3 carriers during the first post-transplantation year. Furthermore, MC simulation showed that CYP3A5*1/*3 genotype may be indirectly associated, through Tac C_0_/D, with lower eGFR values in the late post-transplantation period. Therefore, if CYP3A5 genotyping becomes an integral part of routine practice in kidney transplantation, it could be an additional tool, alongside Tac IPV, to identify patients at risk of graft function deterioration in the late period after Tx.

## Figures and Tables

**Table 1:** Characteristics of the patient population.

Gender recipient (Male/Female)	66/37 (64%/36%)
Age of recipient (years) *	39 (31–47)
Donor type (Living/Deceased)	74/29 (72%/28%)
Body mass (kg) at 6th month	72.0 (62.5–80.0)
BMI (kg/m^2^) at 6th month	23.73 (22.21–26.11)
CRE (µmol/L) at 6th month	134 (113–162)
eGFR (mL/min/1.73 m^2^) at 6th month	47.48 (40.32–57.09)
BUN (mmol/L) at 6th month	7.60 (5.80–9.80)
Dialysis vintage (months)	7.00 (2–21.5)
CYP3A5 genotype: *1/*1;*1/*3;*3/*3;	0/15/88
Acute graft rejection (yes)	5 (4.9%)
Delayed graft function (yes)	13 (12.6%)
Diabetes mellitus (yes)	17 (16.5%)
Hypertension (yes)	83 (80.6%)
Ischemic heart disease (yes)	4 (3.9%)
Tac IPV (%)	22.51 ± 9.71 21.21 (15.03–27.67)

Data are expressed as absolute number and percentage or median and interquartile range (only for Tac IPV did we add mean and standard deviation). * age at the beginning of the study; BMI—body mass index; CRE—serum creatinine concentration; eGFR—estimated glomerular filtration rate; BUN—blood urea nitrogen; Tac—tacrolimus; IPV—intrapatient variability.

**Table 2:** Pharmacokinetic and clinical parameters of interest in relation to Tac formulation.

Parameter of Interest	Tac-TD (*n* = 78)	Tac-OD (*n* = 25)	Test and Significance
eGFR (mL/min/1.73 m^2^) at 6th month	49.90 ± 16.67	47.77 ± 11.58	Z = −1.766; *p* = 0.077
eGFR (mL/min/1.73 m^2^) during 13–36 months	50.14 ± 16.80	48.28 ± 16.43,	Z = −1.337; *p* = 0.181
Acute graft rejection (yes)	4/78	1/25	χ^2^ = 0.052, *p* = 0.819
CYP3A5*1/*3 genotype	11/78	4/25	χ^2^ = 0.055, *p* = 0.815
Mean Tac C_0_/D during 6–12 months (ng/mL/mg)	1.91 ± 1.07	1.81 ± 1.00	Z = −0.604; *p* = 0.546
Tac IPV (%)	22.49 ± 9.5521.29 (14.26–28.17)	22.57 ± 10.4020.44 (17.96–25.26)	Z = −0.192; *p* = 0.848

Data are expressed as absolute number and percentage or mean and standard deviation eGFR—estimated glomerular filtration rate; BUN—blood urea nitrogen; Tac—tacrolimus; Tac-TD—twice-a-day formulation; Tac-OD—once-a-day formulation; IPV—intrapatient variability.

**Table 3:** Multivariate regression analysis of factors influencing the eGFR values from 13 months and up to 36 months after Tx.

MODEL*	B (CI for B)	Std. Error	Beta	Sig. ^1^	R ^2^ (%)	Sig. ^2^
Multivariate Analysis/ Predicators						
Constant	11.256 (8.205–14.306)	1.555	/	<0.001	57.4	<0.001
eGFR at 6 months (mL/min/1.73 m^2^)	0.764 (0.729–0.799)	0.018	0.706	<0.001		
Tac IPV% (absolute value)	−0.103 (−0.165–(−)0.040)	0.032	−0.054	0.001		
Sex (male)	1.439 (0.297–2.581)	0.582	0.042	0.014		
Age (years)	−0.011 (−0.062–0.040)	0.026	−0.007	0.664		
Mean C_0_/D from 6–12 months (ng mL^−1^/mg)	1.676 (1.061–2.290)	0.313	0.096	<0.001		
Acute graft rejection (yes)	−10.112 (−12.664–(−)7.559)	1.301	−0.133	<0.001		

B—unstandardized regression coefficient; CI—95 % confidence interval; Beta—standardized regression coefficient; R squared—proportion of the variance around the mean of the eGFR that is explained by the present model; Std. Error—Standard error; ^1^ The significance of the predictor within a proposed model; ^2^ The significance of the proposed model itself.

**Table 4:** Input values of the independent parameters used for simulation.

Parameter	Values
eGFR at 6th month post-transplantation (base value for eGFR)	30–44 mL/min/1.73 m^2^
	45–59 mL/min/1.73 m^2^
Tac IPV	15–29.99%
	30–59.99%
CYP3A5 genotype (as mean C_0_/D during 6–12 months)	CYP3A5*1*/3 = 1.30 ± 0.54 ng/mL/mg
	CYP3A5*3*/3 = 1.92 ± 0.98 ng/mL/mg
Sex	Male = 1
	Female = 0
Acute rejection episode within the first post-transplantation year	Yes = 1
	No = 0

## Data Availability

The data presented in this study are available on reasonable request from the corresponding author.

## References

[1] Shuker N., van Gelder T., Hesselink D.A. (2015). Intra-patient variability in tacrolimus exposure: Causes, consequences for clinical management. Transpl. Rev..

[2] Thongprayoon C., Hansrivijit P., Kovvuru K., Kanduri S.R., Bathini T., Pivovarova A., Smith J.R., Cheungpasitporn W. (2020). Impacts of High Intra- and Inter-Individual Variability in Tacrolimus Pharmacokinetics and Fast Tacrolimus Metabolism on Outcomes of Solid Organ Transplant Recipients. J. Clin. Med..

[3] Jouve T., Fonrose X., Noble J., Janbon B., Fiard G., Malvezzi P., Stanke-Labesque F., Rostaing L. (2020). The TOMATO Study (Tacrolimus Metabolization in Kidney Transplantation): Impact of the Concentration-Dose Ratio on Death-censored Graft Survival. Transplantation.

[4] Birdwell K.A., Decker B., Barbarino J.M., Peterson J.F., Stein C.M., Sadee W., Wang D., Vinks A.A., He Y., Swen J.J. (2015). Clinical Pharmacogenetics Implementation Consortium (CPIC) Guidelines for CYP3A5 Genotype and Tacrolimus Dosing. Clin. Pharmacol. Ther..

[5] Stefanović N.Z., Cvetković T.P., Jevtović-Stoimenov T.M., Ignjatović A.M., Paunović G.J., Veličković R.M. (2015). Investigation of CYP 3A5 and ABCB1 gene polymorphisms in the long-term following renal transplantation: Effects on tacrolimus exposure and kidney function. Exp. Ther. Med..

[6] Thölking G., Fortmann C., Koch R., Gerth H.U., Pabst D., Pavenstädt H., Kabar I., Hüsing A., Wolters H., Reuter S. (2014). The tacrolimus metabolism rate influences renal function after kidney transplantation. PLoS ONE.

[7] Shuker N., Shuker L., van Rosmalen J., Roodnat J.I., Borra L.C., Weimar W., Hesselink D.A., van Gelder T. (2016). A high intrapatient variability in tacrolimus exposure is associated with poor long-term outcome of kidney transplantation. Transpl. Int..

[8] Stefanović N.Z., Veličković-Radovanović R.M., Danković K.S., Catić-Djordjević A.K., Damnjanović I.D., Mitić B.P., Cvetković M.B., Cvetković T.P. (2020). Insight into the potential influence of inter- and intra-individual variability of tacrolimus exposure on graft function decline in three-year period following kidney transplantation. Farmacia.

[9] Bonate P.L. (2001). A brief introduction to Monte Carlo simulation. Clin. Pharmacokinet..

[10] Catić-Đorđević A., Pavlović I., Pavlović D., Stefanović N., Mikov M., Cvetković T., Veličković-Radovanović R. (2018). Evaluation of gender-based limited sampling methods for tacrolimus exposure after renal transplantation using the Monte Carlo simulation. Pharmazie.

[11] Law J.P., Borrows R., McNulty D., Sharif A., Ferro C.J. (2021). Early renal function trajectories, cytomegalovirus serostatus and long-term graft outcomes in kidney transplant recipients. BMC Nephrol..

[12] Levey A.S., Coresh J., Greene T., Stevens L.A., Zhang Y.L., Hendriksen S., Kusek J.W., Van Lente F. (2006). Using standardized serum creatinine values in the modification of diet in renal disease study equation for estimating glomerular filtration rate. Ann. Intern. Med..

[13] Hart A., Smith J.M., Skeans M.A., Gustafson S.K., Wilk A.R., Castro S., Foutz J., Wainright J.L., Snyder J.J., Kasiske B.L. (2020). OPTN/SRTR 2018 Annual Data Report: Kidney. Am. J. Transplant..

[14] Borra L.C., Roodnat J.I., Kal J.A., Mathot R.A., Weimar W., van Gelder T. (2010). High within-patient variability in the clearance of tacrolimus is a risk factor for poor long-term outcome after kidney transplantation. Nephrol. Dial. Transplant..

[15] Larpparisuth N., Pongnatcha T., Panprom P., Promraj R., Premasathian N., Vongwiwatana A. (2021). High Intra-Patient Variability in Tacrolimus Exposure Calculated over a Long Period Is Associated with De Novo Donor-Specific Antibody Development and/or Late Rejection in Thai Kidney Transplant Patients Receiving Concomitant CYP3A4/5 Inhibitors. Ther. Drug Monit..

[16] Gonzales H.M., McGillicuddy J.W., Rohan V., Chandler J.L., Nadig S.N., Dubay D.A., Taber D.J. (2020). A comprehensive review of the impact of tacrolimus intrapatient variability on clinical outcomes in kidney transplantation. Am. J. Transplant..

[17] Schütte-Nütgen K., Thölking G., Steinke J., Pavenstädt H., Schmidt R., Suwelack B., Reuter S. (2019). Fast Tacrolimus Metabolizers at Risk—It is Time for a C/D Ratio Calculation. J. Clin. Med..

[18] Kuypers D.R.J. (2020). Intrapatient Variability of Tacrolimus Exposure in Solid Organ Transplantation: A Novel Marker for Clinical Outcome. Clin. Pharmacol. Ther..

[19] Zhang X., Lin G., Tan L., Li J. (2018). Current progress of tacrolimus dosing in solid organ transplant recipients: Pharmacogenetic considerations. Biomed. Pharmacother..

[20] van Gelder T., Meziyerh S., Swen J.J., de Vries A.P.J., Moes D.J.A.R. (2020). The Clinical Impact of the C0/D Ratio and the CYP3A5 Genotype on Outcome in Tacrolimus Treated Kidney Transplant Recipients. Front. Pharmacol..

[21] Morris T.P., White I.R., Crowther M.J. (2019). Using simulation studies to evaluate statistical methods. Stat. Med..

[22] Sablik K.A., Clahsen-van Groningen M.C., Hesselink D.A., van Gelder T., Betjes M.G.H. (2018). Tacrolimus intra-patient variability is not associated with chronic active antibody mediated rejection. PLoS ONE..

[23] Kim E.J., Kim S.J., Huh K.H., Kim B.S., Kim M.S., Kim S.I., Kim Y.S., Lee J. (2021). Clinical significance of tacrolimus intra-patient variability on kidney transplant outcomes according to pre-transplant immunological risk. Sci. Rep..

[24] Thölking G., Schmidt C., Koch R., Schuette-Nuetgen K., Pabst D., Wolters H., Kabar I., Hüsing A., Pavenstädt H., Reuter S. (2016). Influence of tacrolimus metabolism rate on BKV infection after kidney transplantation. Sci. Rep..

[25] Nowicka M., Górska M., Nowicka Z., Edyko K., Edyko P., Wiślicki S., Zawiasa-Bryszewska A., Strzelczyk J., Matych J., Kurnatowska I. (2019). Tacrolimus: Influence of the Posttransplant Concentration/Dose Ratio on Kidney Graft Function in a Two-Year Follow-Up. Kidney Blood Press Res..

[26] Hesselink D.A., Bouamar R., Elens L., van Schaik R.H., van Gelder T. (2014). The role of pharmacogenetics in the disposition of and response to tacrolimus in solid organ transplantation. Clin. Pharmacokinet..

[27] Brunet M., van Gelder T., Åsberg A., Haufroid V., Hesselink D.A., Langman L., Lemaitre F., Marquet P., Seger C., Shipkova M. (2019). Therapeutic Drug Monitoring of Tacrolimus-Personalized Therapy: Second Consensus Report. Ther. Drug Monit..

[28] Khan A.R., Raza A., Firasat S., Abid A. (2020). CYP3A5 gene polymorphisms and their impact on dosage and trough concentration of tacrolimus among kidney transplant patients: A systematic review and meta-analysis. Pharm. J..

[29] Rodrigo E., Segundo D.S., Fernández-Fresnedo G., López-Hoyos M., Benito A., Ruiz J.C., de Cos M.A., Arias M. (2016). Within-Patient Variability in Tacrolimus Blood Levels Predicts Kidney Graft Loss and Donor-Specific Antibody Development. Transplantation.

[30] Mendoza Rojas A., Hesselink D.A., van Besouw N.M., Baan C.C., van Gelder T. (2019). Impact of low tacrolimus exposure and high tacrolimus intra-patient variability on the development of de novo anti-HLA donor-specific antibodies in kidney transplant recipients. Expert Rev. Clin. Immunol..

[31] Vanhove T., Vermeulen T., Annaert P., Lerut E., Kuypers D.R.J. (2016). High Intrapatient Variability of Tacrolimus Concentrations Predicts Accelerated Progression of Chronic Histologic Lesions in Renal Recipients. Am. J. Transplant..

[32] Thölking G., Schütte-Nütgen K., Schmitz J., Rovas A., Dahmen M., Bautz J., Jehn U., Pavenstädt H., Heitplatz B., Van Marck V. (2019). A Low Tacrolimus Concentration/Dose Ratio Increases the Risk for the Development of Acute Calcineurin Inhibitor-Induced Nephrotoxicity. J. Clin. Med..

[33] Whalen H.R., Glen J.A., Harkins V., Stevens K.K., Jardine A.G., Geddes C.C., Clancy M.J. (2017). High Intrapatient Tacrolimus Variability Is Associated With Worse Outcomes in Renal Transplantation Using a Low-Dose Tacrolimus Immunosuppressive Regime. Transplantation.

[34] Stefanović N.Z., Veličković-Radovanović R.M., Danković K.S., Mitić B.P., Paunović G.J., Cvetković M.B., Cvetković T.P. (2020). Combined Effect of Inter- and Intrapatient Variability in Tacrolimus Exposure on Graft Impairment Within a 3-Year Period Following Kidney Transplantation: A Single-Center Experience. Eur. J. Drug Metab. Pharmacokinet..

[35] Hariharan S., McBride M.A., Cherikh W.S., Tolleris C.B., Bresnahan B.A., Johnson C.P. (2002). Post-transplant renal function in the first year predicts long-term kidney transplant survival. Kidney Int..

[36] Knight R. (2016). Intrapatient variability in tacrolimus exposure—A useful tool for clinical practice. Transpl. Int..

[37] Giza P., Ficek R., Dwulit T., Chudek J., Woźniak I., Więcek A., Kolonko A. (2020). Number of Regularly Prescribed Drugs and Intrapatient Tacrolimus Trough Levels Variability in Stable Kidney Transplant Recipients. J. Clin. Med..

[38] Stifft F., Stolk L.M., Undre N., van Hooff J.P., Christiaans M.H. (2014). Lower variability in 24-h exposure during once-daily compared to twice-daily tacrolimus formulation in kidney transplantation. Transplantation.

[39] Wu M.J., Cheng C.Y., Chen C.H., Wu W.P., Cheng C.H., Yu D.M., Chuang Y.W., Shu K.H. (2011). Lower variability of tacrolimus trough concentration after conversion from prograf to advagraf in stable kidney transplant recipients. Transplantation.

